# Synaptic Changes in AMPA Receptor Subunit Expression in Cortical Parvalbumin Interneurons in the Stargazer Model of Absence Epilepsy

**DOI:** 10.3389/fnmol.2017.00434

**Published:** 2017-12-22

**Authors:** Nadia K. Adotevi, Beulah Leitch

**Affiliations:** Department of Anatomy, Brain Health Research Centre, School of Biomedical Sciences, University of Otago, Dunedin, New Zealand

**Keywords:** absence epilepsy, stargazin, AMPA receptor, parvalbumin, feed-forward inhibition, somatosensory cortex

## Abstract

Feedforward inhibition is essential to prevent run away excitation within the brain. Recent evidence suggests that a loss of feed-forward inhibition in the corticothalamocortical circuitry may underlie some absence seizures. However, it is unclear if this aberration is specifically linked to loss of synaptic excitation onto local fast-spiking parvalbumin-containing (PV^+^) inhibitory interneurons, which are responsible for mediating feedforward inhibition within cortical networks. We recently reported a global tissue loss of AMPA receptors (AMPARs), and a specific mistrafficking of these AMPARs in PV^+^ interneurons in the stargazer somatosensory cortex. The current study was aimed at investigating if cellular changes in AMPAR expression were translated into deficits in receptors at specific synapses in the feedforward inhibitory microcircuit. Using western blot immunolabeling on biochemically isolated synaptic fractions, we demonstrate a loss of AMPAR GluA1–4 subunits in the somatosensory cortex of stargazers compared to non-epileptic control mice. Furthermore, using double post-embedding immunogold-cytochemistry, we show a loss of GluA1–4-AMPARs at excitatory synapses onto cortical PV^+^ interneurons. Altogether, these data indicate a loss of synaptic AMPAR-mediated excitation of cortical PV^+^ inhibitory neurons. As the cortex is considered the site of initiation of spike wave discharges (SWDs) within the corticothalamocortical circuitry, loss of AMPARs at cortical PV^+^ interneurons likely impairs feed-forward inhibitory output, and contributes to the generation of SWDs and absence seizures in stargazers.

## Introduction

Absence epilepsy is the most common early-onset epilepsy, accounting for about 10–17% of all pediatric epilepsies (Matricardi et al., 2014). It is a non-convulsive, generalized genetic epilepsy characterized by sudden, brief loss of consciousness, which can occur 100s of times a day (Berg et al., 2010). Due to the disruptive nature of the seizures, brought on by the many periods of abrupt ‘*absence*,’ some affected children exhibit academic difficulties, psychosocial problems, as well as an increased risk of injury during physical activities (Wirrel et al., 1996; Caplan et al., 2008). In order to reduce these poor outcomes, pharmacological treatment with anti-seizure medication is recommended to reduce the frequency of seizures (Matricardi et al., 2014). Despite years of extensive research into seizure mechanisms, this has not translated into the development of novel anti-epileptic drugs (AEDs) with increased efficacy for treatment (Loscher, 2017). As such, ethosuximide, which was introduced almost 60 years ago (Zimmerman and Burgemeister, 1958), specifically for the treatment of absence seizures (Goren and Onat, 2007), is still the optimal initial monotherapy recommended for treatment of childhood absence epilepsy. However, according to the most recent randomized control trial conducted in accordance with the treatment guidelines of the International League Against Epilepsy (ILAE), ethosuximide treatment failure occurs in 55% of patients during the first year of treatment (Glauser et al., 2013), which could be due to ethosuximide’s broad mechanism of action involving the reduction of excitation (Leresche et al., 1998; Crunelli and Leresche, 2002). In order to develop safe and targeted patient-specific AEDs, there is a need to identify the different cellular and molecular mechanisms underlying absence seizures, taking into account possible variations in genetically-different patients.

Data from human and animal studies have established that absence seizures are associated with spike-wave discharges (SWDs), which arise from aberrant hypersynchronous activity within the corticothalamocortical (CTC) network (Snead, 1995; McCormick and Contreras, 2001). The hypersynchronous oscillations appear to be a consequence of distinct aberrations in different microcircuits of the corticothalamic (CT) and thalamocortical (TC) nodes, which shift the balance between excitatory and inhibitory synaptic transmission in neural circuits. There appear to be multiple mechanisms through which this imbalance can occur. Altered glutamatergic excitation, mainly due to altered receptor function, has been implicated in epileptogenesis in many experimental models (Meldrum et al., 1999). In line with this, perampanel, a non-competitive antagonist of AMPA receptors (AMPARs) (Hanada et al., 2011) that mediates most of the fast glutamatergic excitatory transmission in the brain (Seeburg, 1993), is used in the treatment of some focal and generalized seizures (Kim et al., 2016; Trinka et al., 2016). However, in trials with well-characterized genetic rodent models of absence epilepsy, perampanel, as well as other AMPAR antagonists, have been found to be ineffective against absence seizures (Kamiński et al., 2001; Jakus et al., 2004; Hanada et al., 2011; Citraro et al., 2017). This could indicate a potential difference in the AMPAR-mediated excitatory mechanisms underlying the generation of absence seizures. In earlier studies using the well-established stargazer mutant mouse model of absence epilepsy, we demonstrated a selective loss of excitatory AMPARs specifically at CT synapses onto inhibitory reticular thalamic nuclei (RTN) interneurons (Barad et al., 2012). This underlies reduced AMPAR excitatory currents in these inhibitory neurons (Menuz and Nicoll, 2008). A specific loss in CT-RTN excitation, leading to impaired feed-forward inhibition of thalamic relay nuclei, has also been demonstrated in the absence epileptic Gria4 knockout mouse, which lacks the AMPAR GluA4 subunit (Paz et al., 2011). Together, these studies suggest that a failure of feed-forward inhibitory motifs, due to an impaired glutamatergic AMPAR-mediated excitatory activation of inhibitory neurons, may be key to the generation of SWDs in some absence epilepsy seizure models.

Given that SWDs appear to be initiated in the somatosensory cortex (Meeren et al., 2005; Polack et al., 2007), we turned our attention to the stargazer somatosensory cortex to determine if a similar impairment in feed-forward inhibition is present within specific cortical networks. In the cortex, stargazin, the protein mutated in stargazers (Letts et al., 1998), is selectively expressed in inhibitory neurons, predominantly in parvalbumin-containing (PV^+^) neurons (Maheshwari et al., 2013; Tao et al., 2013), which mediate feed-forward inhibition. We previously demonstrated that there is no change in the density of cortical PV^+^ inhibitory neurons, but there is a decrease in the dendrite:soma ratio of GluA1/4 AMPAR expression in these PV^+^ neurons in stargazers in comparison to non-epileptic (NE) littermates (Adotevi and Leitch, 2016), which could indicate dendritic mistrafficking of these AMPARs to the synapse of PV^+^ neurons, and potential dysregulation of cortical feed-forward inhibition. This finding is consistent with another study, which demonstrated that a mistrafficking of GluA4-AMPARs in PV^+^ neurons could result in cortical interneuron dysfunction, and underlie seizure exacerbation following application of NMDA receptor (NMDAR) antagonists (Maheshwari et al., 2013). These findings suggest that in the stargazer mutant mouse, a downregulation of AMPAR-excitation onto feed-forward inhibitory neurons in the CTC circuitry may underlie seizure activity. However, these studies do not specifically analyze AMPAR expression at identified synapses.

Hence, the aim of our current study was to determine the relative expression of AMPAR at synapses in epileptic stargazers compared with their non-epileptic littermates. Biochemical fractionation was performed to isolate the subcellular components of micropunched tissue from the full depth of the somatosensory cortex of stargazer and NE control littermates. The fractions were processed by western blotting for a comparative analysis of total synaptic AMPAR GluA1–4 expression. The relative expression levels of GluA1–4 at excitatory synapses onto cortical PV^+^ interneurons, was then analyzed using double post-embedding immunogold-cytochemistry with antibodies against PV and AMPAR GluA1–4 to determine changes in AMPAR receptor numbers at synapses of PV^+^ neurons.

## Materials and Methods

### Animals

Experimental procedures were performed on 9–12 week-old male epileptic stargazer (stg/stg) and NE (+/stg, +/+) control littermates. These mice were offspring of stargazer breeding stock obtained from the Jackson Laboratory (Bar Harbor, ME, United States), and raised at University of Otago’s Animal Resource Unit. Mice were maintained on a 12 h light/dark cycle, with access to food and water *ad libitum*. Mice genotypes were confirmed by tail DNA based on guidelines of Jackson Laboratory. All experiments were carried out according to protocols approved by the University of Otago Animal Welfare Office and Ethics Committee.

### Biochemical Fractionation of Cortical Tissue

**FIGURE 1:**
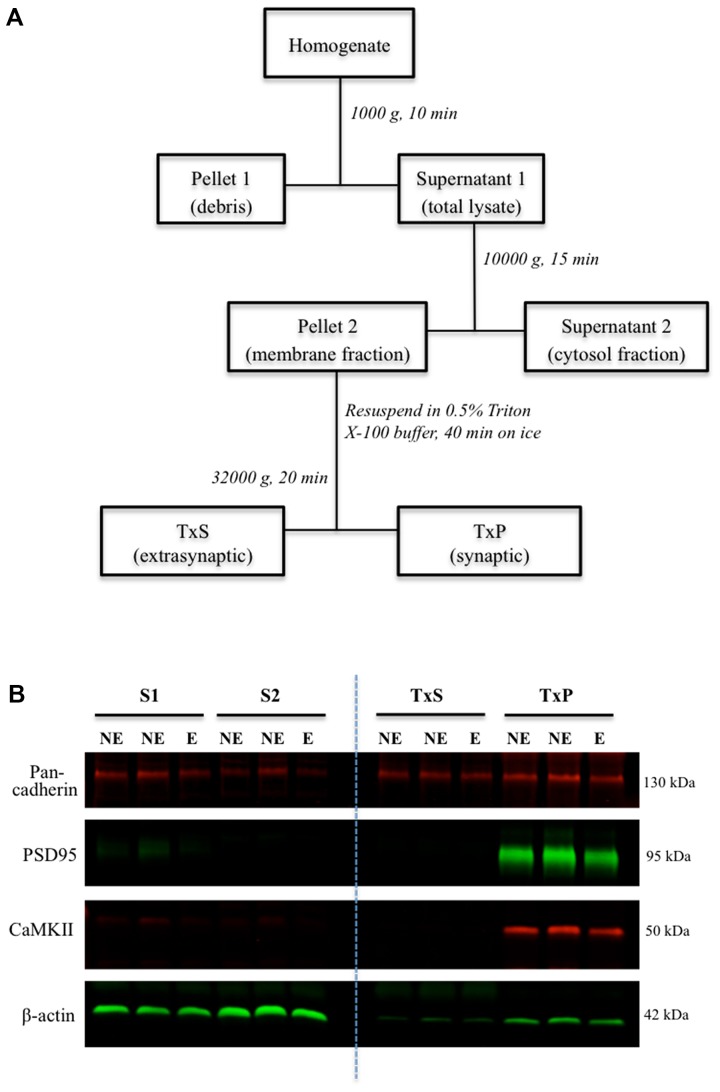
Subcellular fractionation and western blot of fractions. **(A)** Schematic flowchart representation of the biochemical fractionation procedure. TxS is the supernatant fraction containing Triton X-100 soluble extrasynaptic membrane, and TxP is the pellet fraction containing the Triton X-100 insoluble synaptic membrane. **(B)** Representative western blot of subcellular fractions of the somatosensory cortex. Images showing bands corresponding to control markers; pan-cadherin, PSD95, CaMKII and β-actin in the different fractions.

Following cervical dislocation of mice, brains were extracted and snap frozen on dry ice. Brains were sectioned in a freezing cryostat (Leica CM1950, Wetzlar, Germany) into 250 μm coronal sections, which were thaw-mounted onto glass slides. Micropunched tissue taken from the full depth of the somatosensory cortex was homogenized in ice-cold fractionation buffer (10 mM Tris, 320 mM sucrose, 1 mM EDTA, pH 7.4) supplemented with PMSF and protease inhibitor (Sigma, P8340), using sterile plastic pestles coupled with sonication. Synaptic fractions were isolated using a previously described multi-step centrifugation protocol (**Figure 1A**) (Davies et al., 2007; Barad et al., 2017), which relies on the insolubility of synaptic membranes in Triton X-100. Briefly, homogenates were centrifuged at 1000 *g* for 10 min to pellet debris and yield a supernatant. The supernatant was centrifuged at 10000 *g* for 15 min to pellet the cell membrane (membrane fraction). The membrane fraction was resuspended in homogenization buffer containing 0.5% Triton X-100 and incubated on ice for 40 min, and then centrifuged at 32000 *g* for 20 min to yield the pelleted synaptic membrane (TxP) and a supernatant containing extrasynaptic membranes (TxS). TxP was resuspended in homogenization buffer (50 mM Tris, 2 mM EDTA, 3% SDS, pH 6.8). TxS was precipitated with acetone overnight, followed by centrifugation at 3000 *g* for 15 min, and finally resuspended in homogenization buffer for western blotting.

### Western Blotting of Fractions

Western blot analysis of tissue fractions was carried out as previously described (Adotevi and Leitch, 2016). Briefly, 10 μg of protein per sample was separated on 8.5% SDS-PAGE gels, and then transferred onto nitrocellulose membranes for immunoblotting. Protein expression was probed with antibodies against each of the AMPAR subunits; GluA1 (Millipore, AB1504), GluA2 (Millipore, MAB397), GluA3 (Abcam, AB40845) and GluA4 (Millipore, AB1508), as well as fraction markers; CaMKII (Santa Cruz, 13082), Pan-cadherin (Cell Signalling, 4068), PSD-95 (Synaptic Systems, 124 011), and β-actin (Abcam, AB8226). A commercially available protein standard (Novex Sharp Pre-stained Protein Standard; Life Technologies, LC5800) was included as a reference protein molecular weight ladder. Membranes were imaged with the Odyssey Imaging System (LI-COR Biosciences, Lincoln, NE, United States). Relative intensities of the protein bands were measured, with normalized intensities calculated relative to the mean expression of the NE control mice.

### Post-embedding Immunogold Electron Microscopy

Tissue samples were processed and analyzed according to previously established protocols from our group (Barad et al., 2012; Shevtsova and Leitch, 2012; Seo and Leitch, 2015). Mice were anesthetized with sodium pentobarbital (60 mg/kg, Pentobarb 300), and then sacrificed by transcardial perfusion with 4% paraformaldehyde and 0.1% glutaraldehyde in 0.1 M Sorensen’s phosphate buffer (PB). Brains were extracted and further post-fixed overnight at 4°C, before sectioning into 250 μm coronal sections on a vibratome (Leica VT1200, Nussloch, Germany). Tissue trimmed from the full depth of the somatosensory cortex were cryoprotected by slow infiltration in increasing concentrations of sucrose. These were then slam-frozen (Leica KF80, Vienna, Austria), followed subsequently by freeze-substitution and resin embedding (Lowicryl HM20) in an automatic freeze-substitution machine (Leica AFS). Ultra-thin sections were cut and mounted onto formvar-coated nickel grids for immunolabeling. Grid sections were incubated in blocking buffer (10% NGS in TBST, pH 7.4) for 2 h, followed by overnight double-labeling with primary antibodies against the AMPAR GluA1, GluA2/3 (Millipore, AB1506), GluA4 subunits and parvalbumin (Swant, 235), and finally incubated in gold-conjugated to 10 nm goat anti-rabbit and 20 nm goat anti-mouse secondary antibodies (British Biocell, Cardiff, United Kingdom) to identify GluA1–4 and PV with the 10 and 20 nm gold particles respectively. Grids were stained with uranyl acetate and lead citrate, and imaged with the Phillips CM100 transmission electron microscope (Phillips/FEI Corporation, Eindhoven, Holland). Images were analyzed with Image J (NIH, United States), with a comparative analysis of protein expression conducted on tissue from stargazers and control NE mice processed in parallel. Excitatory synapses were identified based on their prominent post-synaptic density (PSD) and asymmetric morphology (Peters et al., 1991). The length of each labeled PSD of an identified PV^+^ cell was measured. Gold particles located within a 30 nm distance from the labeled PSD of PV^+^ neurons were regarded as synaptic, and counted as synaptic GluA1–4 subunits, for comparison between stargazer and NE mice. Density of AMPAR GluA1–4 labeling was obtained by dividing the total number of immunogold particles by the length of the PSD. Antibody specificity was confirmed by omission of primary antibodies and also by preadsorption of primary antibodies with their respective control antigens (used 5x in excess), which were commercially available from the manufacturers of the primary antibodies, namely: parvalbumin recombinant for PV (Swant); GluA1 control peptide for AB1504 (Millipore, AG360); GluA2/3 control peptide for AB1506 (Millipore, AG305); and GluA4 control peptide for AB1508 (Millipore, AG306). Imaging and data analyses were performed with the experimenter blinded to the genotypes. EM grids were coded by an independent non-experimenter, with genotypes revealed only after completion of all quantitative analyses.

### Data Analysis

Data are presented as mean ± standard error of the mean (SEM). Statistical tests were performed using GraphPad Prism 7.0 (GraphPad Software, United States). Comparative analysis of differences in AMPAR expression levels between stargazers and their NE control littermates were assessed by unpaired Mann–Whitney *U*-test, with statistical significance set at *p* < 0.05.

## Results

### Reduced AMPAR GluA1–4 Expression in Stargazer Cortical Synaptic Fractions

**FIGURE 2:**
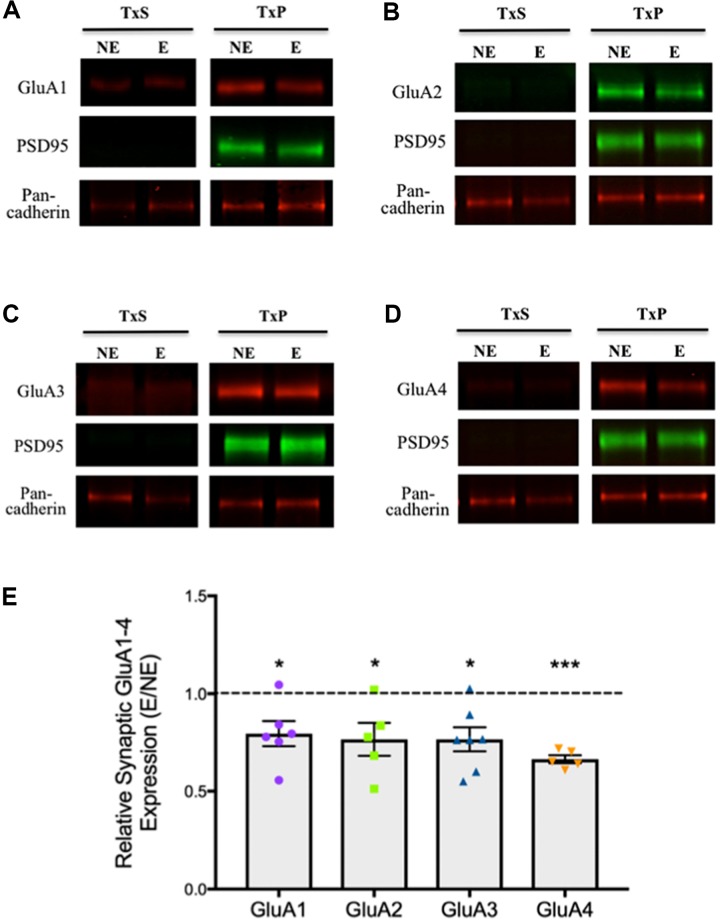
Relative synaptic expression of GluA1–4 in the somatosensory cortex. **(A–D)** Representative immunoblots showing extrasynaptic and synaptic AMPAR subunit expression; **(A)** GluA1, **(B)** GluA2, **(C)** GluA3 and **(D)** GluA4, with the loading markers PSD95 and Pan-cadherin. **(E)** Scatter-plot with bar graphs showing a significant decrease in AMPAR GluA1 (0.79 ± 0.07, *n* = 6, *p* < 0.05), GluA2 (0.77 ± 0.08, *n* = 5, *p* < 0.05), GluA3 (0.77 ± 0.06, *n* = 7, *p* < 0.05), GluA4 (0.66 ± 0.02, *n* = 5, *p* < 0.01) expression in stargazer (E) synaptic fractions relative to NE control littermates after normalization against Pan-cadherin. Symbols ‘^∗^’ indicate *p* value significant digits (^∗^*p* < 0.05, ^∗∗∗^*p* < 0.005).

To analyze relative expression of AMPARs at synapses in epileptic stargazers compared to NE littermates, somatosensory cortex tissue was separated into subcellular components by biochemical fractionation (**Figure 1A**). First, we validated the methodology by confirming the purity of the isolated fractions using western blotting and immunolabeling for synaptic specific protein markers including CaMKII and PSD-95, which are components of the PSD (Petersen et al., 2003). We also immunolabeled cell adhesion protein pan-cadherin (Beesley et al., 1995) and the cytoplasmic protein actin (Schubert and Dotti, 2007). Immunopositive bands corresponding to the markers were detected at their manufacturer-specified molecular weights (42, 50, 95, and 130 kDA respectively). As expected, PSD95 and CaMKII, were enriched in synaptic fractions (TxP), with very low levels in the total lysate (S1). β-actin was present in all fractions except the extrasynaptic membrane (TxS), whereas pan-cadherin was detected in all fractions (**Figure 1B**). To quantify synaptic AMPAR expression in our fractions, we immunolabeled western blots with antibodies against GluA1–4 subunits, which are all expressed in the somatosensory cortex (Adotevi and Leitch, 2016). GluA1–4 expression was enriched in synaptic fractions (TxP), with detectable, but low levels in the extrasynaptic fractions (TxS; **Figures 2A–D**). Because neuronal cadherin does not associate with stargazin (Tomita et al., 2003), and its expression is unaltered in stargazers compared to NE controls (Barad et al., 2017), the AMPAR GluA1–4 relative intensities were normalized to their corresponding pan-cadherin protein band. Analysis of relative AMPAR subunit expression levels in TxP fractions showed that expression of all GluA1–4 subunits was significantly decreased in stargazers compared to their NE littermates. Relative synaptic expression of GluA1 was decreased by 21% (*n* = 6, *p* < 0.05, **Figures 2A,E**), 23% in GluA2 (*n* = 5, *p* < 0.05, **Figures 2B,E**), 23% in GluA3 (*n* = 7, *p* < 0.05, **Figures 2C,E**), and 34% in GluA4 (*n* = 5, *p* < 0.01, **Figures 2D,E**) in epileptic stargazers compared to NE littermates. Full length western blots and Supplementary Material are provided in **Supplementary Figures 1**–**3**.

### Loss of AMPAR Expression at Excitatory Synapses onto Cortical PV^+^ Neurons

**FIGURE 3:**
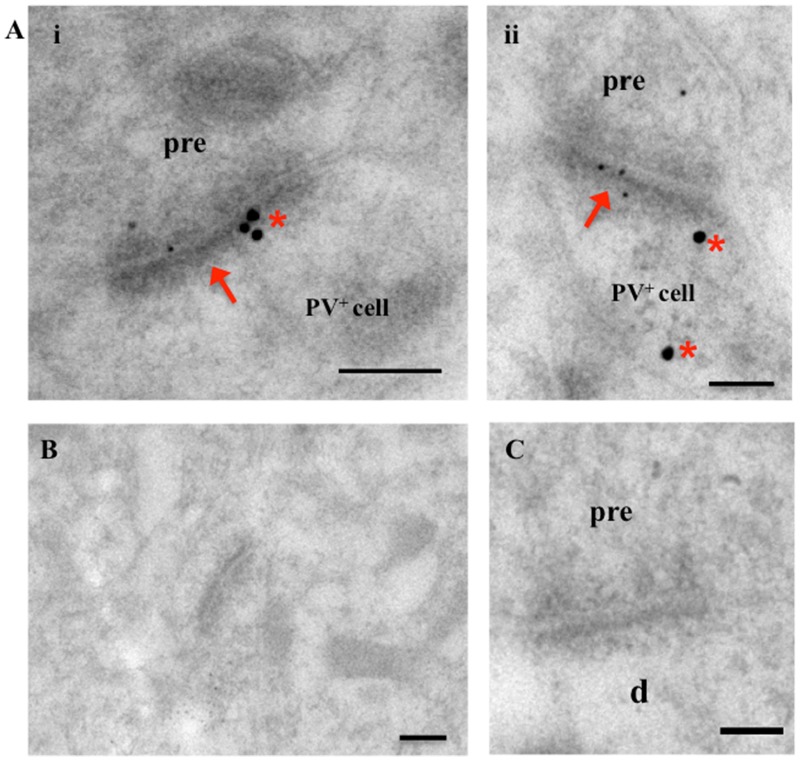
Electron micrographs of excitatory synapses onto PV^+^ interneurons in the somatosensory cortex. **(A)** Images showing excitatory synapses (red arrows) onto PV^+^ neurons (labeled with 20 nm immunogold particles; identified by red asterisk). Synapses show AMPAR subunit labeling (smaller, 10 nm gold particles). **(B,C)** Micrographs of cortical tissue with omission **(B)** and pre-adsorption **(C)** of primary AMPAR and PV antibodies as controls. Absence of gold particles in EM images indicates specificity of immunolabeling in this study. Scale bars = 200 nm.

Having established that AMPARs were significantly reduced in synaptic fractions (TxP), of stargazers compared to NE controls we next wanted to determine if there was a specific loss of AMPARs at synaptic inputs to inhibitory feed-forward neurons in the cortex. We had previously found a loss in the dendrite:soma ratio of GluA1/4-AMPARs in stargazer PV^+^ neurons (Adotevi and Leitch, 2016), which could be an indication of loss of AMPAR-mediated excitation of these neurons. To determine if these changes in AMPAR expression were evident at excitatory synapses onto these PV^+^ neurons, the distribution of GluA1–4 immunogold particles associated with PSDs of these synapses were analyzed in stargazer and control NE mice (*n* = 5 pairs). For each AMPAR subunit, a total of 200 labeled excitatory synapses of immunopositive PV^+^ neurons per subunit per mice group were analyzed. The identification of cortical PV^+^ interneurons was based on the observation of parvalbumin labeling (20 nm gold particles, red asterisk, **Figure 3A**) as described in other studies (Pelkey et al., 2015; Yamasaki et al., 2016). Excitatory synapses onto these PV^+^ neurons were identified by their asymmetrical PSD, as well as, AMPAR immunogold (10 nm) labeling (red arrows, **Figure 3A**). The low levels of background labeling in omission (**Figure 3B**) and preadsorption (**Figure 3C**) control tissue confirmed the specificity of the immunogold labeling observed in this study.

**FIGURE 4:**
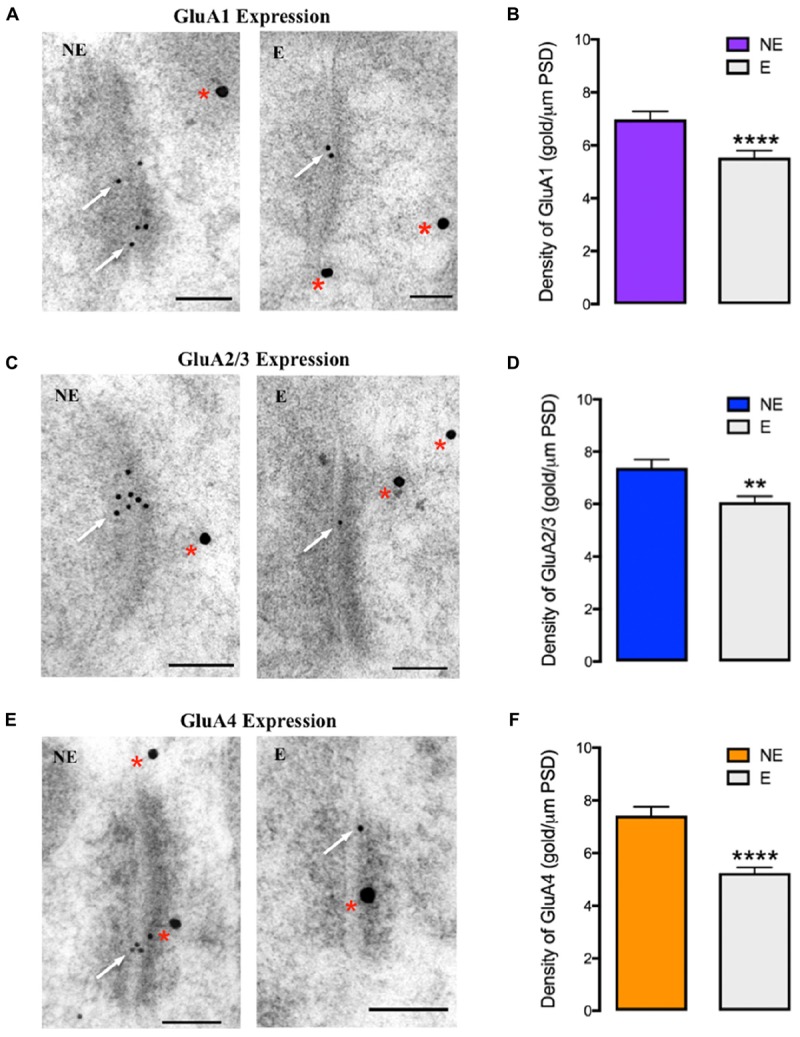
GluA1–4 immunogold labeling at excitatory synapses onto cortical PV^+^ neurons **(A,C,E)** EM micrographs showing labeling of GluA1 **(A)**, GluA2/3 **(C)**, and GluA4 **(E)** at excitatory synapses onto PV^+^ neurons in the somatosensory cortex of NE control and stargazer (E) mice. Off-white arrows indicate gold particles representative of AMPAR subunits’ labeling whereas red asterisks are indicative of PV labeling. **(B,D,F)** Quantitative analysis of GluA1–4 immunogold density per μm PSD length shows reduced GluA1 (NE 6.98 ± 0.306, E 5.54 ± 0.264, *p* < 0.0001), GluA2/3 (NE 7.38 ± 0.320, E 6.06 ± 0.240, *p* < 0.005) and GluA4 (NE 7.42 ± 0.344, E 5.25 ± 0.210, *p* < 0.0001) expression in stargazers (E) compared to control NE mice (*n* = 5 pairs). Scale bars = 100 nm. Symbols ‘^∗^’ indicate *p* value significant digits (^∗∗^*p* < 0.005, ^∗∗∗∗^*p* < 0.0001).

Quantitative analysis of the relative density of synaptic AMPAR subunits (gold particles per μm PSD length) in epileptic and NE littermates revealed statistically significant decreases in GluA1–4 expression at excitatory synapses of stargazer PV^+^ neurons compared to NE mice (**Figure 4**), consistent with previous observations of alterations in total synaptic expression, which could indicate possible defects in AMPAR trafficking. GluA1 expression was reduced by 21% (NE 6.98 ± 0.306, E 5.54 ± 0.264, *p* < 0.0001; **Figures 4A,B**), GluA2/3 by 18% (NE 7.38 ± 0.320, E 6.06 ± 0.240, *p* < 0.005; **Figures 4C,D**) and GluA4 by 29% (NE 7.42 ± 0.344, E 5.25 ± 0.210, *p* < 0.0001; **Figures 4E,F**). There was no statistical difference in average PSD length of excitatory synapses between stargazers and their NE littermates in the somatosensory cortex (NE 322.7 ± 5.235 nm, E 318.0 ± 5.317 nm, *p* = 0.5297), which is in agreement with previous studies in the stargazer cerebellum (Richardson and Leitch, 2005; Meng et al., 2006). In the current study, an antibody that recognizes both GluA2 and GluA3 (anti-GluA2/3; Millipore, AB1506) was used to label synapses immunopositive for GluA2 or GluA3, as immunolabeling with commercial antibodies against either GluA2 or GluA3, which work well in western blot applications, was unsuccessful in electron microscopy (EM) immunogold-cytochemistry applications. Hence, the quantified gold particles represent both subunits. Nonetheless, the similarity in total percentage reductions in total synaptic GluA2 and GluA3 expression, suggest that GluA2 and 3 are decreased in similar proportions at excitatory synapses of stargazer PV^+^ neurons. Notably, previous studies have also reported that GluA2 and GluA3 cortical expression patterns are similar (Sato et al., 1993; Kondo et al., 1997), with GluA3 mostly associated with GluA2 to form GluA2/3 heteromers (Lu et al., 2009).

**FIGURE 5:**
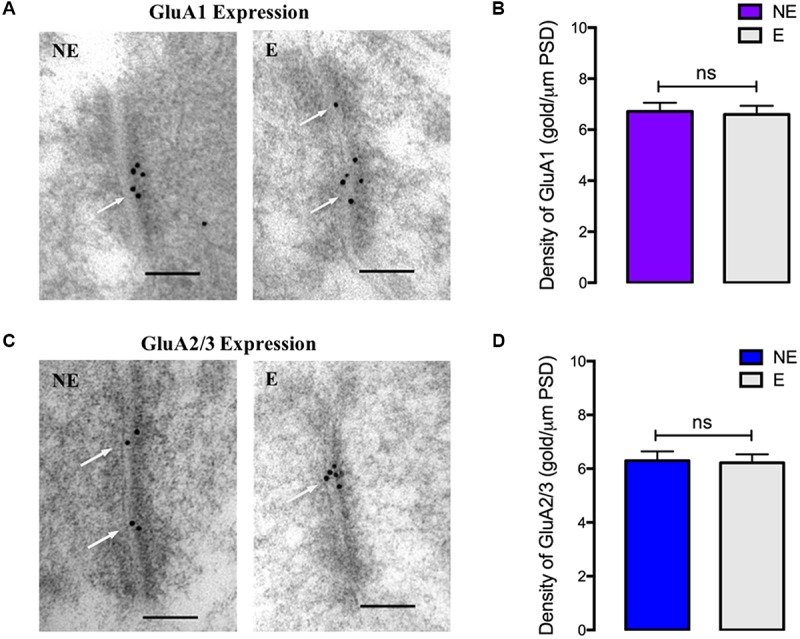
GluA1–3 immunogold labeling at excitatory synapses onto cortical PV negative neurons **(A,C)** EM micrographs showing labeling of GluA1 **(A)** and GluA2/3 **(C)** at excitatory synapses onto PV^-^ neurons in the somatosensory cortex of NE control and stargazer (E) mice. White arrows indicate gold particles representative of AMPAR subunits’ labeling. **(B,D)** Quantitative analysis of GluA1–3 immunogold density per μm PSD length showed no differences in GluA1 (NE 6.71 ± 0.339, E 6.60 ± 0.336, *p* = 0.6156) and GluA2/3 (NE 6.30 ± 0.349, E 6.22 ± 0.317, *p* = 0.7749) expression between stargazers (E) and control NE mice (*n* = 5 pairs). Scale bars = 100 nm.

To determine if the changes in synaptic AMPAR subunit expression were specific to PV^+^ interneurons, quantitative immunogold analysis of the main AMPAR subunits (GluA1–3) expressed by parvalbumin-negative (PV-) neurons (Adotevi and Leitch, 2016) was conducted (**Figure 5**). In contrast to observed reduction of AMPAR expression at excitatory synaptic inputs onto PV^+^ interneurons in stargazers, there was no statistical difference in the relative densities of AMPAR at excitatory synapses in PV negative neurons in stargazers compared to their NE littermates (GluA1: NE 6.71 ± 0.339, E 6.60 ± 0.336, *p* = 0.6156, **Figures 5A,B** and GluA2/3: NE 6.30 ± 0.349, E 6.22 ± 0.317, *p* = 0.7749; **Figures 5C,D**). These data provide evidence that the reduction in AMPAR expression at synapses in the somatosensory cortex of stargazers affects PV^+^ neurons.

## Discussion

In the current study, we analyzed the synaptic expression of AMPAR GluA1–4 subunits in the somatosensory cortex of stargazers compared to their NE control littermates to determine if there is a loss of AMPAR expression at input synapses onto inhibitory interneurons, based on our previous findings suggestive of a dendritic mistrafficking of AMPARs in cortical PV^+^ neurons (Adotevi and Leitch, 2016). We demonstrate for the first time that, in the stargazer cortex there is a reduction of total synaptic levels of GluA1–4 in isolated synaptic fractions analyzed with western blotting, and from EM immunogold analysis, a loss of these AMPAR GluA1–4 subunits at excitatory synapses onto inhibitory PV^+^ neurons. These findings provide evidence of a potential loss of synaptic AMPAR-mediated excitation of cortical inhibitory neuron, which could consequently weaken feed-forward inhibitory output, and underlie the generation of SWDs in stargazers.

The loss of GluA1 and 4 containing AMPARs at PV^+^ neuron synapses is not surprising, given that studies report that PV^+^ neurons predominantly express GluA1 and 4-containing AMPARs (Geiger et al., 1995; Kwok et al., 1997). Furthermore, it has been previously reported that stargazin expression is associated with GluA4 containing PV^+^ neurons in the stargazer cortex (Maheshwari et al., 2013). Given that most cortical GluA4^+^ neurons co-express GluA1 (Kondo et al., 1997), this may also account for the 29 and 21% reductions in GluA4 and GluA1 expression respectively at PV^+^ synapses. Nonetheless, the effects of the stargazin deficit does not seem limited to only GluA1/4-AMPARs, as we also observed a reduction of GluA2/3 expression (GluA2/3 expression was reduced in total synaptic fractions by 23% and at identified PV^+^ synapses by 18%). These subunits are also expressed in PV^+^ neurons (Geiger et al., 1995; Kwok et al., 1997; Wang and Gao, 2010). These results are consistent with findings in the stargazer thalamus, which also shows a loss of GluA2/3-AMPARs at inhibitory RTN synapses (Barad et al., 2012). Thus, taken together, our results indicate a significant loss of AMPARs, predominantly GluA4-containing AMPARs, at synapses onto PV^+^ neurons in the stargazer somatosensory cortex.

Although we observed a significant reduction in AMPAR expression, a proportion of receptors were still present at synapses of stargazer PV^+^ neurons. Other studies on stargazers indicate that total absence of GluA subunits only occurs at synapses of neurons (e.g., granule cells of the cerebellum), which depend exclusively on stargazin as their sole Type I TARP (Chen et al., 2000; Tomita et al., 2003; Fukaya et al., 2005; Vandenberghe et al., 2005). Conversely, in the stargazer thalamus (Barad et al., 2012) and cortex where there is reduced but not complete loss of synaptic AMPARs, this may indicate that other TARPs or alternatively other AMPAR auxiliary proteins such cornichons (Schwenk et al., 2009) could mediate AMPAR trafficking to these synapses in the absence of stargazin. Nonetheless, our results indicate a marked reduction in AMPAR expression at PV^+^ interneuron synapses, which suggests that the potential expression of other auxiliary proteins does not fully compensate for the loss of stargazin in these neurons. Additionally, our data indicate that the loss of AMPAR expression in the stargazer cortex affects PV^+^ neurons since no changes in subunit expression were observed at synaptic inputs onto PV^-^ neurons (other non-parvalbumin inhibitory neurons and excitatory neurons). Hence, our current findings give credence to the proposal that reduced AMPAR-mediated excitation of fast-spiking PV^+^ neurons engenders a subsequent failure of these inhibitory neurons to provide adequate feed-forward inhibition within cortical networks.

The loss of feed-forward inhibition within the CTC circuitry, which is required to prevent runaway excitation, has been suggested as a potential SWD-causing mechanism in different models of absence epilepsy (Sasaki et al., 2006; Paz et al., 2011; Maheshwari et al., 2013; Adotevi and Leitch, 2016). Recent evidence indicates that the loss of excitatory input, particularly to inhibitory PV^+^ interneurons (Maheshwari et al., 2013; Pelkey et al., 2015; Adotevi and Leitch, 2016), could affect inhibitory feed-forward activity and thus render the CTC circuitry susceptible to SWDs. AMPARs primarily drive fast and robust excitatory neurotransmission in the CNS, and previous studies have shown that excitatory inputs onto inhibitory interneurons are often larger in magnitude than equivalent inputs onto neighboring excitatory pyramidal cells (Porter et al., 2001; Gabernet et al., 2005; Cruikshank et al., 2007). PV^+^ neurons depress rapidly once activated by excitatory inputs to mediate “early-onset” feed-forward inhibition of excitatory pyramidal cells, which shortens the temporal “window” for excitation (Lalanne et al., 2016), and thus prevents overexcitation within cortical networks. Indeed, seizures are thought to be a result of neuronal hyperexcitability, due to an imbalance between excitatory and inhibitory transmission (Snead, 1995; Timofeev and Steriade, 2004). Given that in the cortex, stargazin is predominantly expressed in PV^+^ inhibitory interneurons, it is most likely that excitation of pyramidal neurons remains intact. This is further supported by our finding that there is no loss of AMPAR expression at PV negative neurons in the somatosensory cortex, of which the majority are likely to be pyramidal cells. Hence, the loss of GluA1–4 AMPARs in stargazer PV^+^ neurons, demonstrated in this study, could lead to a dysfunction of cortical PV^+^ feed-forward inhibitory microcircuits due to reduced excitation-induced activation of these interneurons. This is in agreement with the suggestion that a reduction of synaptic excitation of PV^+^ neurons, but not excitatory neurons, may result in a decrease in feed-forward inhibition (Paz and Huguenard, 2015). The impaired feed-forward inhibition would be insufficient to overcome the recurrent excitation of cortical pyramidal neurons (Douglas et al., 1995), and thus create a hyperexcitable cortex susceptible to SWDs.

Although other researchers have proposed an alternative mechanism wherein NMDARs are capable of mediating the excitation of inhibitory neurons in the absence of AMPARs, and even upregulate inhibitory function (Lacey et al., 2012), studies of these NMDARs in stargazer CTC oscillatory activity continue to provide contradictory results (Aizawa et al., 1997; Lacey et al., 2012; Maheshwari et al., 2013; Barad et al., 2017). For instance, in a very recent study, even in the absence of synaptic AMPAR expression in the stargazer RTN, there was no corresponding compensatory increase in synaptic NMDAR expression (Barad et al., 2017). In addition, all the conclusions from the different studies do suggest that changes in NMDAR expression and function in inhibitory networks, if any, are a consequence of preceding changes in AMPAR expression and function, highlighting a critical role for the loss of AMPAR-mediated activation of PV^+^ feed-forward interneurons in SWDs in stargazers. Another mechanism for a reduction in excitatory drive onto PV neurons could be a reduction in the number of excitatory synapses onto these neurons (as occurs in schizophrenia, Chung et al., 2016). Although, a quantification of excitatory synapse numbers onto PV neurons was not conducted in this study, it is possible that the stargazer mutation also impacts on synapse density.

In summary, the loss of synaptic AMPAR-mediated excitation of PV^+^ neurons suggests that disinhibition in cortical networks, due to insufficient feed-forward inhibition of excitatory neurons, may underlie seizure activity in the stargazer mutant mouse. In this way, impaired feed-forward inhibition seems to be a SWD-generating factor in some cases of absence epilepsy. Of note, stargazin expression has also been reported in a smaller number of other cortical GABAergic inhibitory interneurons, e.g., somatostatin-expressing (SOM^+^) neurons (Tao et al., 2013). Hence, it is possible that AMPAR expression may also be altered in these interneurons, and thus epileptic activity in stargazer is associated with a reduction in all types of GABAergic inhibition. Given that feed-forward inhibition is primarily mediated by PV^+^ neurons (Freund and Katona, 2007; Paz and Huguenard, 2015), future studies using methodologies such as DREADDs technology to selectively manipulate the action of cortical inhibitory interneurons could elucidate the dynamics of feed-forward inhibition in absence seizures. And thus, substantiate the proposal that the specific targeting and regulation of excitatory activation of inhibitory interneurons could be a potential seizure-suppressing mechanism in some absence epilepsy patients.

## Author Contributions

BL conception, hypothesis development and design of the research, secured the funding. NA helped with research design and conducted experiments. Analyses, interpretation of the results were conducted jointly. Both authors contributed to manuscript writing and approved the final version.

## Conflict of Interest Statement

The authors declare that the research was conducted in the absence of any commercial or financial relationships that could be construed as a potential conflict of interest. The reviewer HM and handling Editor declared their shared affiliation.

## Supplementary material

FIGURE S1 Western blot analysis of subcellular isolated fractions of the mouse somatosensory cortex. Blot images showing bands corresponding to control markers; pan-cadherin, PSD95, CaMKII and β-actin in the different fractions. S1 is the supernatant 1 containing total cortical tissue lysate, S2 is the supernatant containing the cytosol fraction, TxS is the supernatant fraction containing Triton X-100 soluble extrasynaptic membrane, and TxP is the pellet fraction containing the Triton X-100 insoluble synaptic membrane.

FIGURE S2 Western blot analysis of GluA1-4 expression in extrasynaptic (TxS) and synaptic (TxP) fractions. Images showing expression of **(A)** GluA1, **(B)** GluA2 and GluA4, **(C)** GluA3 and the loading control markers pan-cadherin and PSD95.

FIGURE S3 Western blot analysis of CaMKII expression in synaptic fractions. **(A)** Blot images showing expression of CaMKII, PSD95 and Pan-cadherin in isolated subcellular fractions. **(B)** Bar graph showing no difference in CaMKII expression in synaptic (TxP) fractions (highlighted in yellow) in stargazers compared to NE controls after normalization with pan-cadherin (NE 1.00 ± 0.030, E 1.05 ± 0.1176, *p* = 0.1436).
